# Interaction of *Schistosoma mansoni* Sporocysts and Hemocytes of *Biomphalaria*

**DOI:** 10.1155/2012/743920

**Published:** 2012-06-28

**Authors:** D. Negrão-Corrêa, A. C. A. Mattos, C. A. J. Pereira, R. L. Martins-Souza, P. M. Z. Coelho

**Affiliations:** ^1^Departamento de Parasitologia, ICB-UFMG, Avenida Antônio Carlos 6627, 312170-901 Belo Horizonte, MG, Brazil; ^2^Laboratório de Esquistossomose, Centro de Pesquisa René Rachou/FIOCRUZ, Avenida Augusto de Lima 1715, 30190-002 Belo Horizonte, MG, Brazil; ^3^Laboratório de Parasitologia Básica, ICB-UNIFAL, Rua Gabriel Monteiro da Silva 700, 37130-000 Alfenas, MG, Brazil

## Abstract

Human infection by *Schistosoma mansoni* affects more than 100 million people worldwide, most often in populations of developing countries of Africa, Asia, and Latin America. The transmission of *S. mansoni* in human populations depends on the presence of some species of *Biomphalaria* that act as an intermediate host. The compatibility between *S. mansoni* and its intermediate host is influenced by behavioral, physiological, and genetical factors of the mollusc and the parasite. The susceptibility level of the mollusc has been attributed to the capacity of internal defense system (IDS)—hemocytes and soluble components of the hemolymph—to recognize and destroy the parasite, and this will be the center of interest of this paper. The schistosome-resistant *Biomphalaria* can be an alternative strategy for the control of schistosomiasis.

## 1. Introduction

Schistosomiasis is an important health problem that affects over 200 million people worldwide. Among the schistosome species that infect humans, *Schistosoma mansoni* is the most prevalent species causing intestinal and hepatic schistosomiasis in more than 100 million people living mainly in sub-Saharan Africa, the Caribbean, and South America, including Brazil [1, 2]. Although campaigns for schistosomiasis control based on chemotherapy have reduced the morbidity and prevalence of this disease, transmission continues in almost all the areas in which interventions has been attempted. The transmission of *S. mansoni* in human populations has been associated with environmental and socioeconomic conditions, but the presence of susceptible intermediate hosts, consisting of some species of *Biomphalaria*, is obligatory. In Brazil, out of the eleven species *Biomphalaria* [3], only three were found naturally infected by *S. mansoni*: *B. glabrata*, *B. tenagophila*, and *B. straminea* [4].

The development of *S. mansoni* inside the intermediate host starts immediately after the active penetration of the snail by the miracidium, a swimming ciliated larva, through the exposed snail tegument. After penetration, the parasite undergoes morphological and physiological changes, being transformed into primary sporocyst (or mother sporocyst) that remains in the fibromuscular tissue of the host's cephalopodal region, near the penetration site. After 2-3 weeks, primary sporocysts generate secondary ones (or daughter sporocysts), which migrate from the cephalopodal musculature to the digestive glands or hepatopancreas of the mollusc, where their germinative cells can generate the cercariae [5–7]. The susceptibility level of different *Biomphalaria* species or strains to infection with the same lineage of *S. mansoni* can be very diverse, and it is a determinant of vectorial competence.

The compatibility between *S. mansoni* and its intermediate host is influenced by behavioral and physiological factors of the mollusc. However, the susceptibility level of *Biomphalaria* to *S. mansoni* is also determined by the genetic differences of the molluscs, as well as by the genetic constitution of *Schistosoma* [8, 9]. Newton [10, 11] demonstrated that the susceptibility of *B. glabrata* snail to *S. mansoni* depends largely upon genetic factors. Later, these results were corroborated by Richards [12], who demonstrated that the resistance character, acquired at the maturity phase, is determined by a single dominant gene with mendelian inheritance. The genetic dominance of the resistance character was also confirmed in crossbreeding with the susceptible and resistant strain of *B. tenagophila* [13, 14]. One of the factors that influence susceptibility, and that may be genetically determined, is the activity of the snail internal defense system (IDS).

The *Biomphalaria* IDS is composed of soluble components of hemolymph and circulating cells, termed hemocytes, which work in association during the snail responses against infectious agents [15]. In snails, circulating hemocytes, especially the phagocytic cell population, are the principal line of cellular defense involved in destruction of *S. mansoni* larvae inside the intermediate host [16–23]. However, there is experimental evidence that soluble elements of the hemolymph participate in the protective mechanism against pathogens in many invertebrates [24–27]. Soluble components of the hemolymph can interact directly with pathogenic agents producing toxic substances or lytic peptides, or indirectly through mediator molecules for recognition of the pathogen or hemocyte activators [22, 28–32]. In *Biomphalaria*, hemocytes circulating in hemolymph or fixed in tissues are mainly produced by a well-defined region located between the pericardium and the posterior epithelium of the mantle cavity, called the amebocyte producing organ (APO) [33]. However, there is some evidence [34–37] that *B. glabrata* hemocytes may have multicentric origin and sites with proliferation of hemocytes were detected also at the saccular portion of the renal tubules and in the ventricular cavity of the heart. 

The existence of a cellular defense mechanism deployed by molluscs against trematode infection was initially suggested by the finding of histological reactions around parasite sporocysts [10]. Histopathological analysis of *S. mansoni*-infected *Biomphalaria* showed that hemocyte infiltration around parasite larvae was faster and stronger in snail strains that are more resistant to parasite infection [23, 38]. The confirmation of hemocyte participation in *S. mansoni*-sporocyst control was provided by experiments that transferred the APO from resistant to susceptible snail strains. In these experiments the APO recipient snails were able to control *S. mansoni* infection better than the respective controls [39, 40].

The effector mechanisms by which hemocytes are able to kill trematode larvae are partially dependent on the capability of these cells to recognize sporocyst tegument molecules, leading to parasite encapsulation and cellular activation, that result in production of highly toxic metabolites of oxygen and nitrogen associated with parasite killing [41–45]. In this context, a better knowledge of the interactions between the parasite tegument and snail hemocytes is essential for understanding the snail susceptibility to *S. mansoni* infection. This is needed in order to propose new strategies for parasite transmission control. During the last few years, our research group has used the experimental model of *S. mansoni* infection in *B. tenagophila* of Taim strain to explore this interaction and the results are discussed below.

## 2. *Schistosoma mansoni* Infection in **** *Biomphalaria tenagophila* of Taim Strain

*Biomphalaria tenagophila* is the second major intermediate host of *S. mansoni* in Brazil. Snails of this species are well distributed through the southeast and south states of Brazil, from Bahia to Rio Grande do Sul, being responsible for disease transmission in the state of São Paulo and for several disease foci in the states of Santa Catarina, Minas Gerais, Rio de Janeiro, and Rio Grande do Sul [4, 46, 47]. Besides Brazil, *B. tenagophila* also occurs in Argentina, Peru, Bolivia, Paraguay, and Uruguay [4]. The susceptibility levels of *B. tenagophila* collected from different geographic areas to infection with the same lineage of *S. mansoni* are diverse. As far as *B. tenagophila* is concerned, the geographic lineage isolated at the biological reservoir in Taim (Rio Grande do Sul, Brazil), designated as Taim strain, is absolutely resistant to *S. mansoni* [13, 48, 49], and the resistance of this *B. tenagophila* lineage has been explored in our laboratory, where we study the possible mechanisms of the parasite's destruction. Experimental infections in *B. tenagophila* Taim have shown that *S. mansoni* miracidia are able to penetrate this snail strain; however the parasites induce an intense cellular infiltration in the infection site leading to parasite destruction within a few hours of infection [23], suggesting an important participation of the IDS on determination of resistance to *S. mansoni* in *B. tenagophila* Taim. The importance of hemocytes in the parasite control was confirmed by experiments that transferred the hematopoietic organ (APO) from snail of Taim strain to *B. tenagophila* susceptible to *S. mansoni* infection. The transference resulted in an absolute resistance against the challenge with *S. mansoni* in receptor snail whose APO transplant was successful [40].

The process of destruction of *S. mansoni* larvae by hemocytes starts with the recognition and encapsulation of the newly penetrated sporocyst. The tegument of *S. mansoni* transforming miracidium is an important interface for molecular communication between the parasite and *Biomphalaria* [50]. In this context, the first step in the activation of this defense mechanism is the recognition of the parasite presence by hemocytes. The tegument of *S. mansoni* sporocyst is composed of highly glycosylated [50–52] molecules that bind to soluble proteins of *B. glabrata* hemolymph in a carbohydrate-dependent manner [50]. Furthermore, it was demonstrated that excretory-secretory glycoproteins from *S. mansoni* sporocysts also bind to hemocytes via carbohydrate binding receptors [53]. Therefore, lectin-carbohydrate binding could mediate the association of hemocytes with the trematode tegument [15, 44, 53], and consequently it could be a determinant factor of *Biomphalaria *susceptibility to *S. mansoni* infection. 

To better understand the interaction of hemocytes with parasite larvae, we used the *in vitro* assay first developed by Bayne et al. [16]. Using this procedure we tested the effect of purified circulating hemocytes plus soluble hemolymph from different *Biomphalaria* species or strain on the axenically transformed primary or secondary sporocysts of *S. mansoni*. 

The data clearly showed that addition of purified hemocytes from resistant snail strains, such as *B. tenagophila* Taim, into culture with primary sporocysts resulted in higher levels of parasite mortality compared to sporocysts cultured with hemocytes from susceptible snail strains, such as *B. tenagophila* Cabo Frio [54]. Moreover, in primary sporocyst cultures containing hemocytes from *B. tenagophila* Cabo Frio, the addition of cell-free hemolymph from *B. tenagophila* Taim resulted in increase of hemocyte binding to parasite tegument and higher mortality rates [54]. Therefore, we demonstrated that high levels of sporocyst mortality were associated with higher number of hemocytes bound to parasite tegument leading to parasite encapsulation [32, 54], experimentally confirming that the ability of hemocytes to recognize the primary sporocyst is related to the resistance of *B. tenagophila* Taim.

Finally, we investigated if lectin-carbohydrate binding could mediate the association of hemocytes from *B. tenagophila* Taim with *S. mansoni* primary sporocysts. Previous work [31] with *S. mansoni* infection in *B. tenagophila* Taim showed that most of the circulating hemocytes recovered from *B. tenagophila* Taim, but not from *B. glabrata *BH or *B*. *tenagophila* Cabo Frio that are susceptible to *S. mansoni* infection, were intensively labeled by FITC-conjugated PNA and WGA lectins, and these labeled cells almost disappeared from the circulation during the first few hours after *S. mansoni* infection. Based on these data we tested, *in vitro*, the participation of N-acetyl-D-glucosamine carbohydrate moieties on the adhesion and destruction of *S. mansoni* sporocysts by hemocytes of *B. tenagophila* Taim. Similarly to the previous data, cultures containing hemocytes plus hemolymph from *B. tenagophila* Taim encapsulated and destroyed over 30% of the *S. mansoni* sporocysts in culture. Interestingly, the addition of N-acetyl-D-glucosamine to culture medium, but not mannose, resulted in significant inhibition of cellular adhesion to the parasite tegument and reduction of parasite mortality to 5% [32]. In conclusion, the data indicate that N-acetyl-D-glucosamine moieties influence the recognition of schistosome primary sporocysts by hemocytes of *B. tenagophila* Taim and implies the mechanism is a determinant of snail resistance against *S. mansoni* infection.

According to Lodes and Yoshino [55]; the general pattern of synthesis and release of protein by primary and secondary sporocysts in culture is quite different, showing that the two sporocyst stages are metabolically different. The study of gene expression profiles of *S. mansoni* daughter sporocysts identified different stage-specific genes, several of which are related to adaptation and development of the parasite in the host [56, 57]. The *in vitro* interaction of axenically transformed* S. mansoni* primary sporocysts or secondary sporocysts obtained from infected snails with IDS components of *B. glabrata* (susceptible) and *B. tenagophila* Taim (resistant) revealed that the secondary sporocysts are less affected by the IDS, mainly of the resistant snail. Secondary sporocysts had fewer cells adhered to the surface, lower mortality, and less surface damage. These results suggest higher resistance of secondary sporocysts to the effector mechanisms of *Biomphalaria* when compared to the primary sporocyst. However, the secondary sporocysts were unable to grow when inoculated into *B. tenagophila* Taim but were able to develop into *B. glabrata* [58]. 

Many authors have found that sporocysts can interfere with snail host reproductive physiology and alter other aspects of the parasite-host interaction, secreting molecules (excretory/secretory products (ESP)), adsorbing *Biomphalaria* antigens when cultivated in the presence of snail molecules, and synthesizing molecules similar to host molecules even in the absence of *Biomphalaria* components [55, 59–65]. Recently, experiments with molecular and biochemistry approaches using ESP or sporocysts and hemocytes from schistosome-susceptible and schistosome-resistant *B. glabrata* demonstrated that the parasite is able to interfere with extracellular signal-regulated kinase (ERK) pathway in susceptible *B. glabrata* [66–68]. Moreover, resistant *B. glabrata* presented differential expression of genes potentially associated with the snail IDS after infection with *S. mansoni* when compared with susceptible strain [69].

The hemocytes from resistant *Biomphalaria* species can recognize, encapsulate, and destroy the sporocysts soon after *S. mansoni* invasion. On the other hand, the ability of the parasite to avoid or disrupt the immune response of the host is fundamental to the establishment of parasite-host compatibility [70]. Similar molecules have been found in *S. mansoni* and *Biomphalaria*, suggesting an evolutionary convergence of molecular expression between parasite and snail host [8, 59, 70–72]. This similarity is important for the escape process of the parasite: molecular mimicry [72–74]. According to Salzet et al. [75], this mechanism can prevent the recognition of the parasite by the host IDS.

These data help us to understand why the snails defense in particular its destruction of primary sporocysts, occurs in the first hours after miracidia penetration. Furthermore, van Die and Cummings [71] and Lehr et al. [72] have suggested that glycans play a role in the parasite molecular mimicry process. Although the evolutionary advantages of this adaptive process for the parasite are well understood, it is not known how this process interferes with schistosomiasis [75] or whether this mechanism could interfere with the snail's resistance mechanisms. Thus, more experiments using daughter sporocysts must be performed to clarify aspects involved with molecular mimicry in the *S. mansoni*/*Biomphalaria *(susceptible and resistant) interaction.
